# Supplementary material for: Dialogue with the public: A catalyst for professional identity formation in medical students
Source: PLoS One. 2025 Jun 2;20(6):e0324506. doi: 10.1371/journal.pone.0324506 (PMC12129212; doi:10.1371/journal.pone.0324506)
Supplement: S1 File — (DOCX) [file pone.0324506.s001.docx]

**Supplementary Material: Summary of Japanese References**

**Reference 5**

**Otani T. (2019).** *Paradigm and Design of Qualitative Study: From Research Methodology to SCAT.* The University of Nagoya Press. *(In Japanese)*

**Summary:**

This book provides a comprehensive overview of qualitative research paradigms and methodologies, with a particular focus on SCAT (Steps for Coding and Theorization). It explains the epistemological foundations of qualitative inquiry and how SCAT serves as a structured yet flexible approach for analyzing qualitative data. The book also discusses the advantages of SCAT in small-sample studies and its compatibility with interpretivist research frameworks.

**What is SCAT?**

In qualitative research, researchers usually collect data through observation or interviews and analyze it. While analyzing qualitative data and formulating theories can be a rewarding experience, it is often a challenging process for novice researchers. Unlike statistical analysis methods in quantitative research, qualitative research does not follow a fixed, universal procedure, making data analysis difficult.

To address this challenge, many researchers apply "codes" to their data as a step toward theorization. However, some find it difficult to generate codes, while others struggle even more with writing theories after coding is completed. These challenges may contribute to the hesitation among researchers interested in qualitative research to fully engage with it.

SCAT was developed to help overcome these difficulties in qualitative data analysis.

SCAT consists of two key procedures:

1. **Four-Step Coding:**
   - The segmented data is written in the leftmost column of the SCAT matrix form, followed by consecutive coding steps:
     1. Identifying noteworthy words or phrases from the text
     2. Paraphrasing the identified words/phrases
     3. Extracting external concepts that account for the paraphrases
     4. Developing themes or constructs considering the context
2. **Storyline and Theory Writing:**
   - After completing steps 1 to 4, a storyline is developed based on the themes. Finally, a theory is formulated from the storyline.

SCAT is applicable even to small-scale datasets and is user-friendly, making it a suitable method for beginners in qualitative research.

The details are also available in English on the following website:

<https://www.educa.nagoya-u.ac.jp/%7Eotani/scat/index-e.html>

**Reference 14**

**Ando, R. (2015).** *A Quest for the Significance of Frequent Job Changes in the Present Age: Construction of Zenzai-doji (Sudhana) Career Model.* *(In Japanese)*

**Summary:**

This dissertation explores the phenomenon of frequent job changes in contemporary society, analyzing career transitions through the lens of the **Zenzai-doji (Sudhana) Career Model**. The study argues that career mobility should be seen as a meaningful and constructive process rather than a sign of instability. By using narrative analysis and case studies, the research highlights how individuals develop professional identities through diverse career experiences. The findings suggest that flexible career paths contribute to personal and professional growth in modern labor markets.

**Reference 15**

**Yamamoto, Y. (2013).** *A Study of the Japanese "Verbal Character" among Learners of Japanese: Exploratory Research on the Formative Process, Function, and Signification of the "Verbal Character" through Narrative Analysis of One Learner of Japanese.* *Scripsimus, University of the Ryukyus, No.22, pp.57-73.* *(In Japanese with English Abstract)*

**Summary:**

This study examines the concept of **"Verbal Character"** among learners of Japanese, focusing on its formative process, functions, and significance. Through narrative analysis of a single learner, the research explores how linguistic identity develops during second-language acquisition. The findings indicate that learners construct their "Verbal Character" based on linguistic, cultural, and social influences. This study contributes to the field of language education by emphasizing the role of personal identity formation in language learning.
