# Supplementary material for: Dialogue with the public: A catalyst for professional identity formation in medical students
Source: PLoS One. 2025 Jun 2;20(6):e0324506. doi: 10.1371/journal.pone.0324506 (PMC12129212; doi:10.1371/journal.pone.0324506)
Supplement: S2 File — (DOCX) [file pone.0324506.s002.docx]

**Supporting Information**

**Storyline and Theoretical Description (English Translation)**

1. **Storyline and Theoretical Description for Participant 3FS**

**<Storyline>**

When asked about her motivation for participating in dialogue-based science communication, the student described both a “desire to re-engage based on value judgments from past experiences” and a “dilemma in engaging with elective curricula driven by intrinsic motivation and compulsory curricula driven by extrinsic motivation.”

Regarding her expectations and anxieties during the preparation period, she reported a sense of “stable motivation.” In terms of post-event satisfaction, the student emphasized the value of interactions with “other presenters who shared common interests” and “others of the same generation.”

The student noted that resolving “expert bias caused by ignorance” led to the development of “empathic understanding toward visitors,” and mentioned the “contagion of interest from the visitors.”

With regard to perceived “paternalism in the compulsory curriculum,” the student felt they developed a “sense of responsibility associated with professional rights.”

In response to prompts about “multifaceted interaction at social intersections,” the student referred to “synergistic effects on their own research received from other presenters with shared interests,” as well as the possibility that “interactions with others of the same generation” became a manifestation of “the strength of weak ties.” She also expressed interest sparked by “informational gaps with presenters who shared neither interests nor generation.”

The student highlighted the difference between “unidirectional learning through books” and “bidirectional learning through dialogue,” emphasizing the importance of “understanding tacit knowledge through informal learning.”

Regarding dialogue with the general public, the student mentioned experiences of “collaborative experiential learning” and “transformation into a mimetic leader through peer education.”

She also described how “empathic understanding derived from being a lower-year student” helped bridge the “informational gap between their presentation and the general public.” Through such interactions, they became more aware of their “social identity as a medical student.”

Concerning identity in daily life, the student noted the “limitations of cross-cultural interaction in everyday settings” and the presence of “homogeneous peer communities,” leading to immersion in “non-elective curricula.”

In relation to “collaboration with people from diverse backgrounds” and “multi-roles of academic and work life,” the student described “discomfort arising from self-objectification,” while also recognizing the limitations caused by “topic selection based on information value and cognitive load” and the need to “avoid discrepancies in evaluation standards.”

With regard to “insights from extraordinary experiences,” the student spoke about “projecting oneself onto the serendipity of beginners,” rooted in empathy for the general public, which led to “a sense of experiential satisfaction.”

Finally, the student affirmed the “significance of extraordinary experiences in non-elective curricula connected to personal identity,” and explained how this led to “recognition of responsibility tied to professional rights” through reflections on “clinical clerkships and medical procedures performed by students.” This experience ultimately contributed to “increased motivation to learn” and prompted a final reflection on her “identity as a beginner in the event.”

**<Theoretical Description>**

-The student’s motivation to participate in dialogue-based science communication stems from a “desire to re-engage based on value judgments formed through prior experiences” and a “dilemma in engagement between elective curricula driven by intrinsic motivation and compulsory curricula driven by extrinsic motivation.”

-Expectations and anxieties during the preparation period do not become problematic, due to “stable motivation.”

Satisfaction is influenced by exchanges with “presenters with shared interests” and “presenters of the same generation.”

-Resolving “expert bias caused by ignorance” leads to “empathic understanding toward visitors” and “interest contagion.”

-Perceived “paternalism in the compulsory curriculum” is partially alleviated through the “realization of responsibility associated with rights.”

-“Multifaceted interaction at social intersections” produces “synergistic effects on the student’s own research” from shared-interest presenters, strengthens “weak ties” through generational commonality, and sparks “interest from informational gaps” with others from different groups.

-The difference between “unidirectional learning through books” and “bidirectional learning through dialogue” is defined as the “understanding of tacit knowledge through informal learning.”

-Dialogue with the general public leads to “collaborative experiential learning” and the experience of “transforming into a mimetic leader through peer education.”

-“Information gaps in student presentations,” from the visitor’s perspective, are bridged by “empathic understanding enabled by their junior status,” which facilitates recognition of their “social identity as a medical student.”

-Awareness of “identity in everyday life” is challenged by the “limits of cross-cultural exchange” and the influence of “homogeneous communities.”

-“Immersion in non-elective curricula,” “collaboration with people of different backgrounds,” and “multi-roles of study and work” result in “discomfort due to self-objectification,” yet remain constrained by the need to “select topics based on information value and cognitive load” and “avoid misalignment in evaluation.”

-“Insights from extraordinary experiences” include “projecting oneself onto the serendipity of beginners” through empathy, leading to “a sense of satisfaction.”

-These insights, especially those related to “clinical practice and students’ medical actions,” evoke “a sense of responsibility tied to professional rights,” contribute to “increased learning motivation,” and prompt reflection on “the student’s beginner identity” during the event.

**2. Storyline and Theoretical Description for Participant 5FS**

**<Storyline>**

When asked about her motivation for participating in dialogue-based science communication, the student cited “cross-cultural exchange with individuals outside the medical school community” as a key driver.

She reflected on the “labeling of medical students by outsiders” that she often experienced in daily life and shared with faculty that participating in this “unexpected opportunity” allowed her to confront such experiences.

The student spoke about “praise disguised as labeling” from non-medical individuals she had encountered in the past and expressed a “desire to assert her current efforts.”

She also expressed surprise at the difference between those past interactions and the current event, where the dialogue was initiated by “genuine interest in her ongoing efforts,” and where the disclosure of those efforts felt “natural.” This experience led her to reflect on how, in daily life, she is subject to evaluations by non-medical acquaintances and alumni from elite high schools, often resulting in feelings akin to “impostor syndrome and self-deprecation.”

Regarding the “attempt to reconstruct identity upon entering university,” the student spoke about the “dilemma of self-disclosure toward non-medical individuals who label medical students” and the experience of “discrepancy between social identity–based behavior and personal identity.” She expressed a sense of resistance toward being evaluated based on social identity.

The student explained how actions based on the feeling of being “uniquely positioned as a medical student” due to clinical training contributed to “overcoming resentment toward academic branding.” She described how “constructing personal identity through clinical training and PIF” enabled her to “recognize the naturalness of personal conversations rooted in individual identity.”

In speaking about her reaction to extrinsic motivation driven by classroom test evaluations, the student emphasized her appreciation of “the experiential value of clinical training.” She became more aware of the fusion between “self-awareness of PIF rooted in the physician–patient relationship” and “her own personal identity,” as well as the significance of “vocational training as distinct from relative evaluation through paper-based testing.”

Although the student reported a “new awareness of others’ interest in her efforts and prior misunderstandings,” she noted that she did not perceive a gap in “patients’ interest in medical students” within clinical settings.

Reflecting on the “enjoyable aspect of this extraordinary experience,” the student described a sense of “returning to the everyday life where one’s daily efforts continue.”

**<Theoretical Description>**

-Motivation for participating in dialogue-based science communication includes “cross-cultural exchange with people outside the medical school community,” often triggered by “unexpected opportunities.”

-Experiences of “being labeled by non-medical individuals” and “praise disguised as labeling” serve as catalysts for “asserting current efforts.”

-Dialogues initiated by “genuine recognition of current efforts” and the “natural act of disclosure” are unexpected for medical students.

-Daily interactions with outsiders or alumni from elite high schools often lead to “impostor syndrome and self-deprecation.”

- The “attempt to reconstruct identity upon entering university” involves challenges stemming from the “dilemma of self-disclosure toward non-medical individuals who label medical students” and the “discrepancy between behaviors based on social identity and one’s personal identity.” These difficulties are further intensified by a “sense of resistance toward being evaluated based on social identity.”

-Actions based on a feeling of being “uniquely positioned as a medical student,” especially rooted in clinical practice, enable students to “overcome resentment toward academic branding.” Clinical experience contributes to the “construction of personal identity,” leading to a sense of “natural conversation based on that identity.”

-Awareness of the “context-dependent fluctuation of identity” supports resistance to “extrinsic motivation driven by classroom test evaluations” and promotes “valuation of lived experience from clinical training.”

-The fusion between “PIF rooted in clinical physician–patient relationships” and “personal identity,” as well as the contrast with “relative evaluations via paper-based tests,” reveals the “importance of vocational training.”

-“Awareness of others’ interest in one’s efforts and previous misunderstandings” does not necessarily lead to discomfort with the “lack of patient interest in students” during clinical training.

-Reflection on “the enjoyment of extraordinary experiences” serves as a contrast to the “return to everyday life with continued effort.” **3. Storyline and Theoretical Description for Participant 5MS**

**<Storyline>**

When asked about his motivation for participating in public science communication, the student described a “desire to produce tangible outcomes within his community before venturing into unfamiliar social spaces.” He viewed this event as an “unexpected opportunity to engage with a domain previously unfamiliar to him.”

In response to questions about “self-interest and hesitation in his actions,” the student explained that “novelty in previously neglected areas” sparked curiosity. Regarding the “continuity and change of motivation,” he cited the “ease and accessibility of telling personal stories,” the “First Penguin effect” in valuing initial action, and the “lack of difficulty in participating” as reasons for continued engagement.

Though the student expressed concerns about “whether others would be interested in him” and “feeling different in the science communication context,” he was surprised by and became aware of the “diversity of visitors and their interests.” Despite encountering “dilemmas in ending conversations due to service capacity and difficulty signaling conversation closure,” he also shared experiences of “cultural resonance through detours into humor shaped by group dynamics.”

When selecting topics during “intergenerational dialogue,” the student found it “effortless to speak based on on-the-job training (OJT) and informal learning.” He reflected on “the application of practical knowledge through conversation,” describing the experience as “an opportunity to re-evaluate his OJT.” He emphasized how “storytelling rooted in lived experience” felt easy and natural.

The student also discussed the features of “intercultural dialogue,” noting the “ease of responding to those who asked questions out of genuine interest” and “the special nature of speaking with active listeners whose curiosity extended beyond mere self-relevance.”

When conversing with “non-healthcare acquaintances,” the student reported selecting topics using a “minimal-effort strategy based on information value and cognitive load.”

Reflecting again on the “extraordinary conversations during an extraordinary experience,” the student described the event as a “transformative learning opportunity,” including a “reexamination of professional settings through conversations with non-professionals,” which served as a “low-burden opportunity for PIF.”

The student spoke about how, in his usual conversations, he was expected to show “spontaneity and careful thought because of patient-centeredness,” and how “trimming content to fit professional contexts” was necessary in occupational communication.

In reflecting on “passive and active learning in peer education,” the student also mentioned “feelings of surprise and concern for younger students,” and the experience of “transmitting knowledge through narrative mentoring.”

When asked about “intersecting identities due to belonging to multiple communities,” the student spoke of “social desirability and extrinsic motivation when selecting topics,” but also revealed a “lack of self-awareness about how he presents his learning and educational context,” and discussed the possibility of “survivor bias in self-assessment” and “generational tunnel vision about change.”

He suggested that his “lack of concern about self-presentation” may mirror the “public’s lack of concern about medical education,” and noted a “dissonance between public interest in students and cooperation during clinical training.” This was accompanied by an “awareness of limited procedural experience” and “anxiety and surprise when dealing with sensitive clinical areas.”

When discussing “serendipity in mentoring,” the student emphasized the “value of experiential learning” and explained how “reexamining gaps between intended and actual practice through reflecting on his specialized education” led to “meaning-making and self-affirmation.”

**<Theoretical Description>**

- Motivation for participating in public science communication includes a “desire to achieve concrete results within one’s own group before engaging with unfamiliar communities,” and is often perceived as an “unexpected opportunity to interact with unfamiliar domains.”

- Self-interest and hesitation are influenced by the “freshness of previously neglected areas.”

- Continuity and change of motivation are shaped by the “ease and accessibility of telling personal stories,” the “First Penguin effect” in valuing early action, and the “low barrier to participation.”

- Concerns about a “lack of interest from others” and “feeling different in a science communication context” are mitigated by the “diversity of visitors and their interests.”

- “Bidirectional communication” leads to dilemmas caused by the “difficulty of signaling the end of service,” but also facilitates “cultural resonance through shared humor and group dynamics.”

- “Topic selection in intergenerational dialogue” is eased by “narration based on informal learning through OJT.” Students reappraise their OJT and describe the experience as a “learning process in applying practical knowledge through conversation.”

- In “intercultural dialogue,” communication is facilitated by the “ease of responding to genuinely curious conversation partners” and the uniqueness of “dialogue with active listeners motivated by curiosity beyond self-relevance.”

- When engaging with “non-healthcare acquaintances,” students select topics based on “minimal effort, cognitive load, and information value.”

- The “extraordinary nature of conversations during the event” serves as “transformative learning,” including a “reappraisal of professional settings through conversations with laypeople,” and provides a “low-burden opportunity for PIF.”

- Daily conversations demand “spontaneity and careful thought due to patient-centered communication,” and require “content trimming for professional appropriateness.”

- Reflections on “peer learning” include “feelings of concern and surprise about junior students” and highlight the importance of “transmitting experience through narrative mentoring.”

- “Intersecting identities from multiple communities” result in “topic selection influenced by social desirability and extrinsic motivation,” a “lack of awareness of how one presents educational experiences,” and the effects of “survivor bias and generational tunnel vision.”

- Students perceive that “indifference to self-presentation” may reflect the public’s indifference toward medical education. They also note a “dissonance between public interest in students and cooperation during training,” alongside “limited experience with procedures” and “anxiety regarding sensitive clinical practices.”

- “Mentoring-based serendipity” and the “value of experiential learning” encourage the “reappraisal of practice gaps through reflecting on specialized education,” which fosters “meaning-making and self-affirmation.”

- Paradoxically, a “low motivation for self-presentation through dialogue” increases “empathy and enjoyment in responding to others.”
