# Supplementary material for: Dialogue with the public: A catalyst for professional identity formation in medical students
Source: PLoS One. 2025 Jun 2;20(6):e0324506. doi: 10.1371/journal.pone.0324506 (PMC12129212; doi:10.1371/journal.pone.0324506)
Supplement: Attachment — Submitted filename: Corections.docx [file pone.0324506.s003.docx]

**CORRECTIONS**

1. Title:

a) The title is quite long and somewhat ambiguous. Consider rewording it for clarity and conciseness. For example: "Dialogue with the Public: A Catalyst for Professional Identity Formation in Medical Students"
b) The phrase "That Somehow Feels Right to Me Now" is informal and might not fit well in an academic setting.

2. Abstract:

a) The abstract states that interviews were conducted with three medical students. This is a very small sample size and might not be sufficient for generalizable conclusions. Consider acknowledging this as a limitation.
b) The abstract describes findings about "Lower-year" and "Upper-year" students but does not provide specific supporting evidence.
c) The phrase "counterintuitively promote" should be replaced with something clearer, e.g., "Unexpectedly enhanced".

3. Introduction:

a) The introduction does not sufficiently justify why dialogue with the general public is a crucial factor in professional identity formation (PIF).
b) The study could benefit from further elaboration on how this research fills a gap in existing PIF literature.

4. Method:

a) Institutional Review Boards (IRBs) usually require signed or documented informed consent. Specify if there was a consent form with a clear explanation of rights.

5. Discussion:

a) The discussion primarily relies on Cruess et al.’s work but does not sufficiently engage with other theoretical models.
b) The comparison between Germany, Japan, and the U.S. is interesting but could be expanded.

6. References:

a) Some citations are incomplete: "Otani T. SCAT: steps for coding and theorization, qualitative data analysis method. English version Last updated 4. Nov. 2015 ed." → Provide the correct DOI or official link.
b) Reference 14 and 15 are in Japanese and may not be accessible to all readers. Consider summarizing key insights in English.
